# Identification of different subtypes of ovarian cancer and construction of prognostic models based on glutamine-metabolism associated genes

**DOI:** 10.1016/j.heliyon.2024.e27358

**Published:** 2024-03-11

**Authors:** Xie Yaqing, Gao Yang, Yang Linlin, Ruan Youqing, Yang Henghui, Yang Ping, Yang Hongying, Wang Shaojia

**Affiliations:** Department of Gynecology, The Third Affiliated Hospital of Kunming Medical University, Yunnan Cancer Hospital, Yunnan Cancer Center, Kunming 650118, China

## Abstract

Ovarian cancer (OC) is common malignant tumor of female reproductive system. Glutamine metabolism-related genes (GMRGs) play a key role in ovarian cancer. Here, available database-- The Cancer Genome Atlas (TCGA), Genotype-Tissue Expression (GTEx), and Gene Expression Omnibus (GEO) databases were applied in our research. OC samples from TCGA were divided into different clusters based on Cox analysis, which filtering GMRGs with survival information. Then, differentially expressed genes (DEGs) between these clusters were intersected with DEGs between normal ovary samples and OC samples, and GMRGs in order to obtain GMRGs-related DEGs. Next, a risk model of OC was constructed and enrichment analysis of risk model was performed based on hallmark gene set. Besides, the immune cells ratio in OC samples were detected via Cell type Identification By Estimating Relative Subsets Of RNA Transcripts (CIBERSORT). Finally, we explored a series of potential biomarkers of OC. In this research, 9 GMRGs-related DEGs were obtained. GMRGs-related DEGs were enriched to canonical Wnt signaling pathway.NKD2, C2orf88, and KLHDC8A, which were significantly associated with prognosis, were retained for risk model construction. Based on the risk model, 18 hallmark pathways with significant difference were enriched. Fifteen types of immune cells (such as iDC, NK CD56dim cells, and neutrophils) enjoying significant difference between these 2 risk groups (high risk group ***vs*.** low risk group) were detected, which indicates possible disparate TME in different metabolic subtypes of ovarian cancer.

## 1 Introduction

Epithelial ovarian cancer (EOC) ranks the 8th leading cause of cancer death in women. Besides, EOC is the 2nd cause of gynecologic cancer death [1].High grade serous ovarian cancer (HGSOC) has attracted our attention more because it is the most common type in clinical practice. To date, there is no validated population screening methods for OC, which is quite different from that of other types of malignancies [2]. Therefore, advanced-stage OC patients are quite common, which contribute to poor survival as well [3]. Detection of BRCA gene mutation may contribute to early prevention of OC because of risk-reducing salpingo-oophorectomy (RRSO) before malignancy transformation and PARPi maintenance therapy may benefit patients with BRCA mutation after surgery and chemotherapy [4]. However, the outcome of OC changes moderately in recent 30 years since its advanced stages during diagnosis and special biological traits. Based on available evidence, the estimated age-standardized (per 100,000 women) incidence will continue to increase substantially in next 30 years as well as motality rate [5]. Therefore, individualized treatment is urgently needed for ovarian cancer patient.

Metabolic reprogramming is an important hallmark of cancer. Nutrients such as glucose, glutamine, protein are involved in this complicated process. This malignant metabolic reprogramming leads to biological process such as cancer cell death resisting, proliferation, metastasis and invasion [6]. Glutamine, which is the most abundant circulating amino acid, provides both carbon and nitrogen sources. It plays important role in reactive oxygen species (ROS) homeostasis as well [7]. Low-invasive OC cells are glutamine independent while its counterpart are markedly glutamine dependent. The activation of STAT3 contributes to this more invasive phenotype [8]. ETS1 promotes ovarian cancer metastasis and invasion in glutamine dependent cells via up-regulation of MMP2 and MMP9 [9]. Down-regulation of glutamine associated enzyme GLUL decrease the proliferation ability of OC cell OVCAR-3 and ES-2 significantly. In this condition, p38MAPK pathway is inhibited markedly [10]. The over-expression of small RNA miR-450a hinder metastasis and invasion of ovarian cancer cell A2780 and SKOV-3. Increased anoikis is present here, which is closely associated with glutamine withdrawal [11]. Accumulating evidence indicates that glutamine metabolism is associated with ovarian cancer regarding to proliferation, invasion and metastasis. Further exploration of glutamine metabolism could be promising in anticancer clinical practice.

In this research, we integrated transcriptome data of OC from TCGA database with normal subject from GTEx of GEO database via bioinformatic methods and constructed a risk model for OC in combination with data from the GEO database to predict patient prognosis. We confirmed biomarkers which are associated with prognosis in OC regarding glutamine metabolism. This may contribute to prognosis prediction in OC patients and promising targets confirmation for future clinical application.

## 2 Results

### 2.1 GMRGs played an important role in OC

In order to figure out the relationship between GMRGs and the progression of the OC, a series of analyses were conducted on the basis of normal ovary samples from the GTEx database and OC patient samples from The Cancer Genome Atlas (TCGA)database. First of all, we removed the batch effect of data from different databases and compared expression of GMRGs between normal ovary samples and their counterparts (Fig. 1A). The result showed that there were 39 GMRGs (such as GLS, TAT, OAT etc.) between the two kinds of samples with significant difference (*p* > 0.05) (Fig. 1B). Next, a PPI network was created to detect the relationships between GMRGs, which indicated that there were strong interactions among GMRGs (Fig. 1C). Following we calculated the mutations of GMRGs on the basis of the somatic mutation data, and the result demonstrated that six genes including GAD2, SLC7A8, UROC1, GLUD2, SLC38A2, and SLC6A14 have a mutation frequency of more than 5%. This result illustrates that these GMRGs are likely to be involved in the progression of OC (Fig. 1D).

**Fig. 1:**
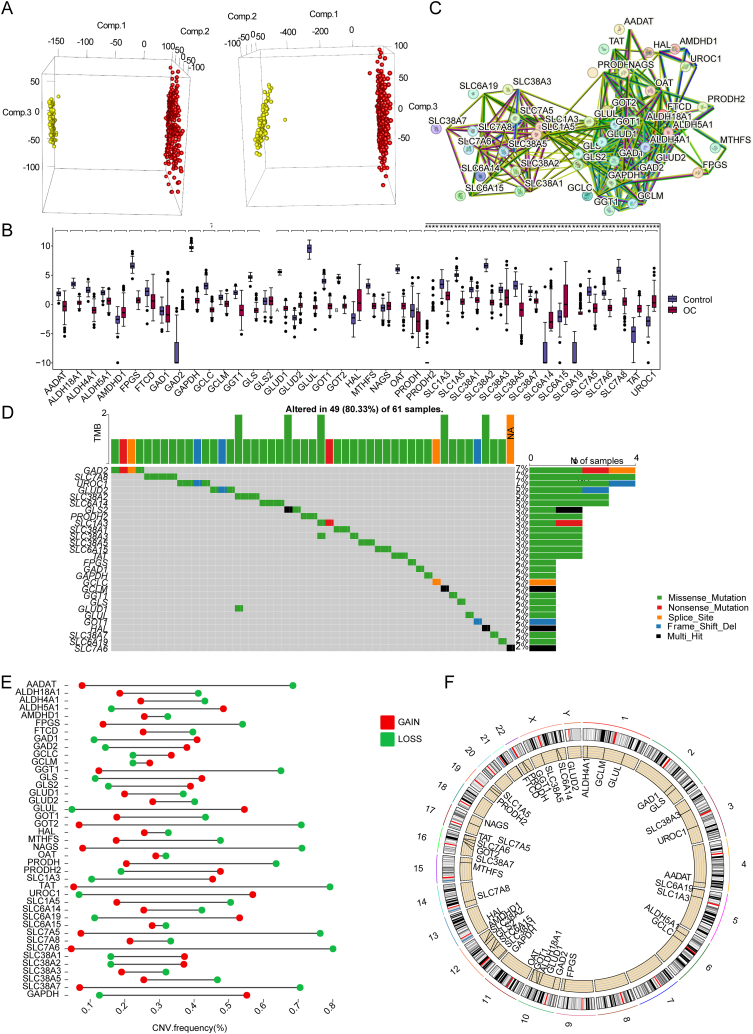
GMRGs played an important role in OC A. Expression level of GMRGs between normal and OC patient samples; B. Detailed information of GMRGs with significant difference between these two kinds of samples; C. Strong interactions among GMRGs showed in PPI network; D. Six genes among these GMRGs presented somatic mutation more than 5%; E. Forty-one genes showed copy number variations; F. Chromosomal location of these 41 GMRGs.

Copy number variations (CNV) is a structural variation in a gene or chromosome, which are mainly reflected in two categories including deletions (loss) and insertions (gain). We got the CNV frequency of 41 GMRGs in samples and found that genes including *GOT2*, *NAGS*, *TAT*, *SLC7A5*, *SLC7A6*, and *SLC38A7* have a high deletion frequencies (more than 0.7%) (Fig. 1E). In addition, we chromosomally localized the 41 GMRGs, the location in the chromosome of them were showed in Fig. 1F. In summary, GMRGs plays a prominent role in the progression of OC.

### 2.2 OC samples were clustered into two groups based on GMRGs with survival correlation

**Fig. 2:**
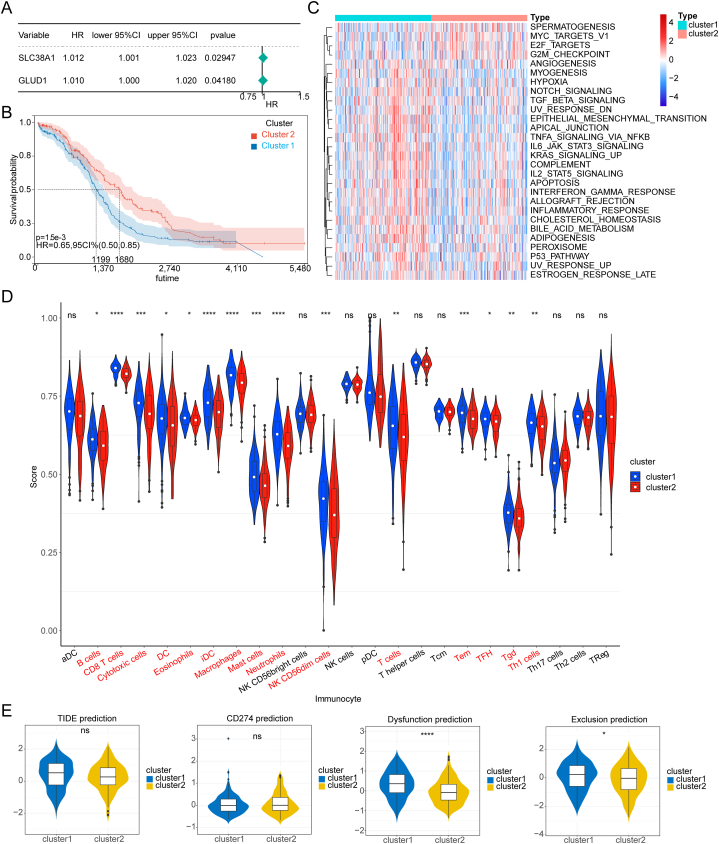
GMRGs based classification of OC patients A. SLC38A1 and GLUD1 were applied to calculate the glutamine metabolic scores of OC patients; B. OC patients were divided into 2 clusters based on scores; C. Significantly different hallmark pathways were confirmed in these 2 clusters; D. Infiltrating immune cells in these 2 clusters; E. Simple micro-immune environment assessment in these 2 clusters.

For the goals of screening GMRGs with a strong relationship with OC, the first step is to perform Univariate Cox analysis based on 41 GMRGs. Two GMRGs including SLC38A1 and GLUD1 were sifted out and applied to calculate the scores of OC patients (Fig. 2A). Finally, OC patients were sorted into 2 clusters (cluster 1*vs*. cluster 2)based on the median of the scores. Next, we performed survival analysis between two clusters and the result indicated that patients in cluster 1 enjoyed better survival than that in cluster 2 (Fig. 2B). Then, we used the ssGSEA algorithm based on 50 hallmark pathways to obtain 28 hallmark pathways which are significantly different between these 2 clusters. It is distinguishable that pathways including SPERMATOGENESIS, MYC TARGETS V1, F2F TARGETS, and G2M CHECKPOINT are up-regulated in cluster 2, and the other pathways are up-regulated in cluster 1 (Fig. 2C). Following proportion of infiltrating immune cells in the samples of normal and OC patients were deduced and we found that 15 infiltrating immune cells such as Eosinophils, iDC, Macrophages, and Neutrophils have a significant difference between two clusters (*p* < 0.0001) (Fig. 2D). Besides, we acquired the TIDE prediction, CD274 prediction, T cell exclusion prediction, and T cell dysfunction prediction of samples between normal and OC patient. The results suggest that both T cell dysfunction prediction and T cell exclusion prediction in cluster 1 are significantly greater than that in cluster 2 (Fig. 2E). Overall, genes in cluster 1 are more likely significantly associated with the development of OC.

### 2.3 Nine GMRGs-related DEGS were obtained by difference analysis

**Fig. 3:**
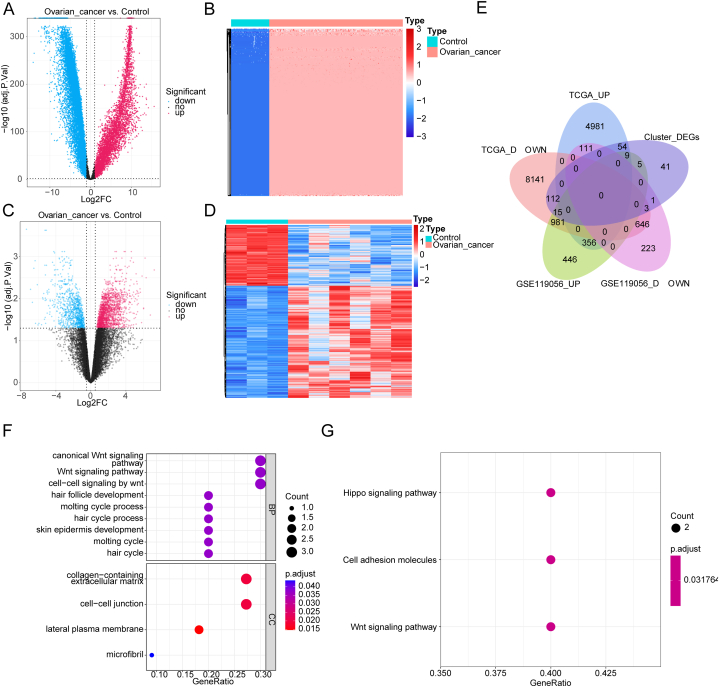
Glutamine metabolic related DEGs in OC and normal samples A-B. DEGs screened out in database (88 normal ovary VS. 379 OC samples); C-D. DEGs screened out in GSE 119056; E. GMRGs-related DEGs set in intersection; F-G. GO and KEGG pathway involved in GMRGs-related DEGs.

Firstly, the transcriptome data of 88 normal ovary and 379 OC samples were downloaded from GTEx and TCGA databases respectively. Then, DEGs between OC samples and their counterparts were recognized by “limma” package. In total, 5511 up-regulated and 9898 down-regulated DEGs from 15409 DEGs were screened out with |log_2_FC| > 0.5 and *adj.p* < 0.05 (Fig. 3A, table S1). The top 100 most significant genes were displayed in Fig. 3B. After, we did the same way between 3 normal ovarian and 6 OC samples from GSE 119056, and between samples from two clusters which classified previously. And 2796 DEGs including 1812 up-regulated and 984 down-regulated DEGs (Fig. 3C and D), and 240 DEGs including 69 up-regulated and 171 down-regulated DEGs **(tables S2 and S3)** were sifted out respectively. In the end, we produced an intersection among 3 kinds of DEGs obtained previously, and the intersection was defined as a GMRGs-related DEGs set. There are 9 up-regulated GMRGs-related DEGs including NKD2, COL9A2, NXPH4, ISM1, WNT10A, CLDN16, C2orf88, GJB2, and MFAP5, and 3 down-regulated GMRGs-related DEGs including KLHDC8A, OMD, and CDH3 in the intersection (Fig. 3E).

In addition, we conducted an enrichment analysis of GMRGs-related DEGs to find enriched functions and pathways in OC samples. The result includes 9 Gene Ontology (GO)- biological progress (BPs), 4 GO- cellular component (CCs), and 3 Kyoto Encyclopedia of Genes and Genomes (KEGG) pathways, and there are 3 GO-BPs including canonical Wnt signaling pathway, Wnt signaling pathway, and cell-cell signaling by Wnt, and 2 GO-CCs including collagen-containing extracellular matrix and cell-cell junction have a high degree of enrichment (Fig. 3F and G).

### 2.4 Classified OC samples into two gene clusters

**Fig. 4:**
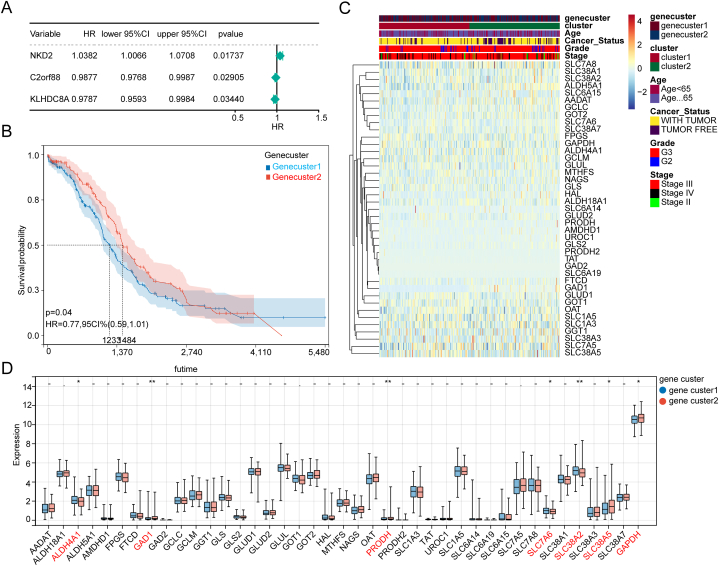
OC Samples classification A.Three survival associated genes were confirmed in 12 GMRGs-related DEGs; B. OC classification based on 3 genes mentioned above was characterized with significantly different survival; C. Holistic information presented in heatmap; D. Seven significantly different GMRGs were confirmed with in these 2 clusters.

We performed Univariate Cox analysis again based on 12 GMRGs-related DEGs, and 3 genes including NKD2, C2orf88, and KLHDC8A which were related with survival were sifted out (Fig. 4A). After, these genes were used to perform ssGSEA to divide OC samples into gene cluster1 including 217 samples and gene cluster2 including 153 samples. Next, a Kaplan–Meier (K-M) survival curve was generated which suggested that the survival of gene cluster 1 is significantly poor than that in gene cluster 2 (Fig. 4B). The heatmap provides a holistic representation of gene expression levels, clinical information, and classification in all samples (Fig. 4C). Besides, we found that there are 7 GMRGs with significant difference including ALDH4A1, GAD1, PRODH, SLC7A6, SLC38A2, and GAPDH between two gene clusters (Fig. 4D).

### 2.5 Construction and performance evaluation of OC risk model

**Fig. 5:**
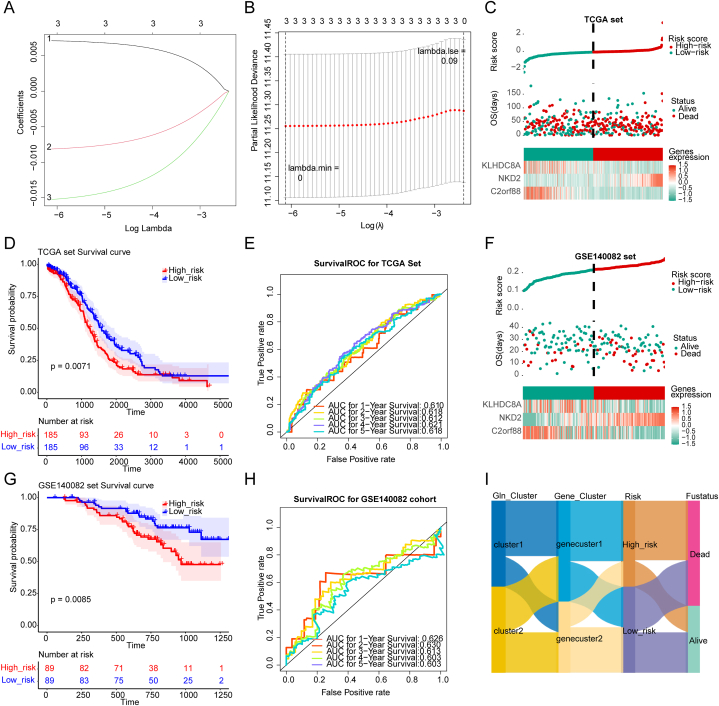
Construction and performance evaluation of OC risk model A-B. Three genes mentioned above in LASSO analysis; C. Risk score calculation based on 3 genes and their expression in high or low risk group; D. Survival probability was significantly lower in high risk group; E.AUC curve was greater than 0.6 in different year interval; G-H. Risk model performance evaluation in validation set; I. Sankey diagram of cluster1/2(SLC38A1 and GLUD1 based score), gene cluster1/2(NKD2, C2orf88, KLHDC8A based evaluation) and risk model.

Least absolute shrinkage and selection operator (LASSO) analysis was used for further filtering of 3 GMRGs-related DEGs which were screened out via Univariate Cox analysis previously, and the result is that 3 genes including NKD2, C2orf88, KLHDC8A remained (Fig. 5A and B). Following, these 3 genes were used for the calculation of risk score, and then we divided the samples from both the training set and validation set into high- and low-risk groups based on the median of risk scores. Next, we assessed the performance of the risk model on the basis of the training group by generating the risk curve, K-M curve, and ROC curve. The risk curve shows that KLHDC8A and C2orf88 are up-regulated in low-risk group, and NKD2 is up-regulated in high-risk group (Fig. 5C). The K-M curve demonstrates that the survival of high-risk group is significantly lower than that of low-risk group (Fig. 5D). Area under the curve (AUC) of 1-, 3-, and 5-years in the receiver operating characteristic (ROC) curve is greater than 0.6 (Fig. 5E). Finally, we used the same methods to verify the performance of already constructed risk model based on the validation set. The results were consistent with the previous ones, which demonstrate that the risk model of OC has a good performance in predicting OC prognosis (Fig. 5F–H). In summary, we twice divided the samples and finally conducted a risk model of OC, the changes in the belonging of samples were illustrated in a Sankey diagram (Fig. 5I)

### 2.6 Clinical correlation and enrichment analyses of the risk model

**Fig. 6:**
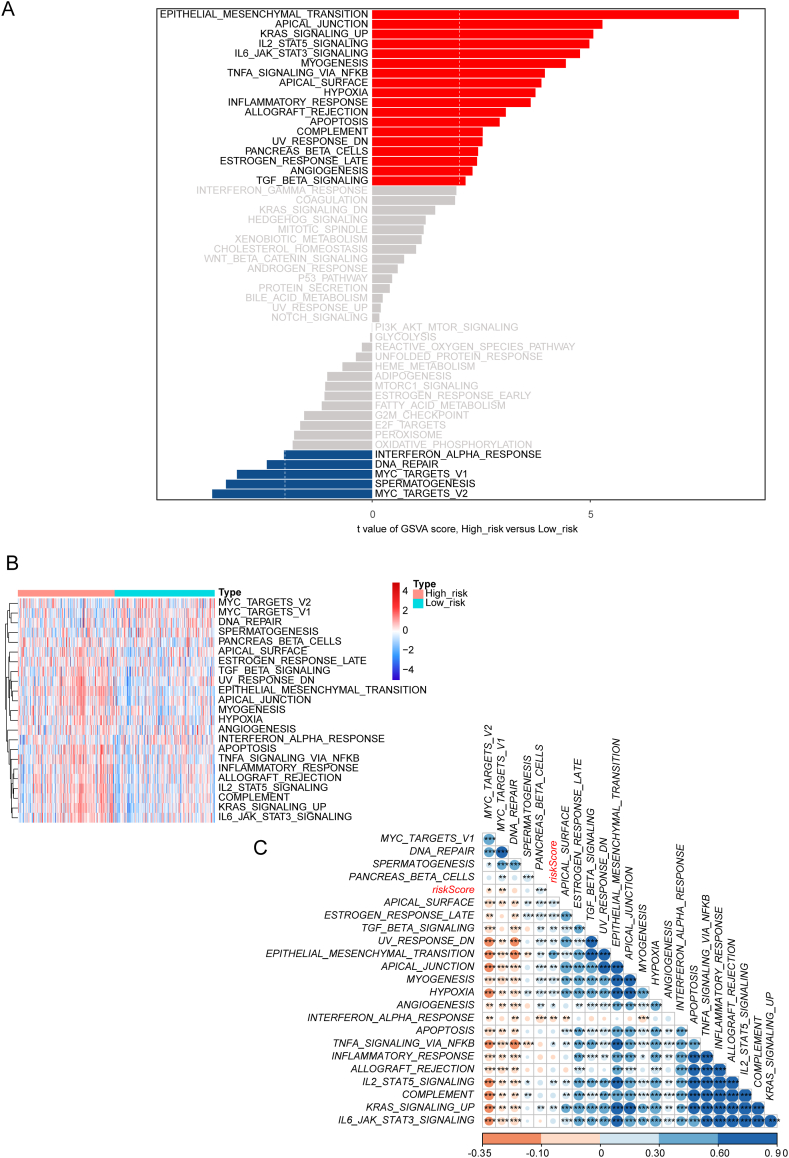
Clinical correlation and enrichment analyses of the risk model A. Enrichment analysis of the risk model and DNA repair pathway is involved; B. Heatmap illustrated the enrichment of hallmark pathways in samples from two risk groups; C. The correlation between risk scores and enriched pathways.

Survival analysis was performed based on different clinical characteristics or survival information of these samples and we found that there is a significant difference between 2 groups (high risk group *vs*. low risk group)in the conditions of age, cancer states, and G staging **(Supplementary fig. 1 A, B). Next, we conducted an enrichment analysis of the risk model on the basis of the hallmark gene set and got** 18 hallmark pathways with significant differences via variance analysis which includes 13 up-regulated pathways in the high-risk group and 5 up-regulated pathways in the low-risk group. Of note, there are two pathways involved in DNA repair which includes UV RESPONSE DN and DNA REPAIR pathway (Fig. 6**A**). The heatmap illustrates the enrichment of hallmark pathways in samples from two risk groups (Fig. 6**B**). Finally, we identified the correlation between risk scores and enriched pathways. The result indicated that the EPITHELIAL MESENCHYMAL TRANSITION pathway has a strong correlation with the risk score (Fig. 6**C**).

### 2.7 There are significant differences in immunology between risk groups

**Fig. 7:**
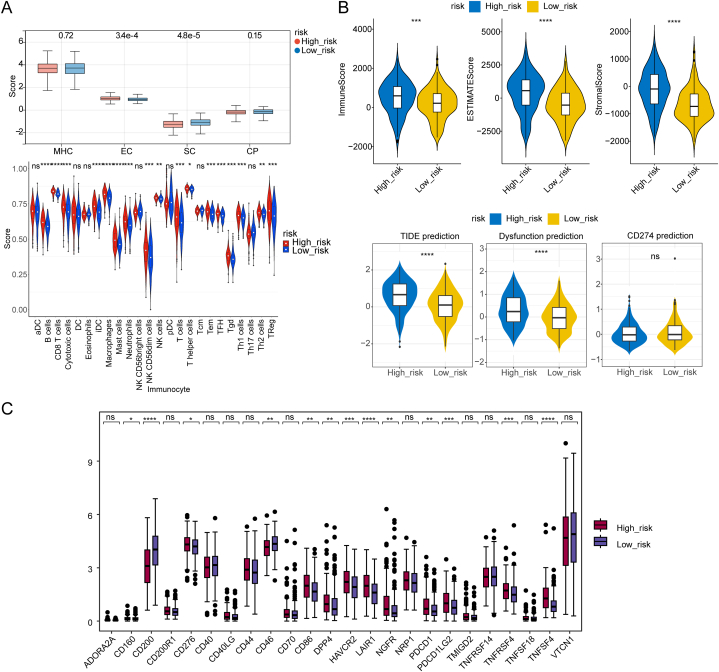
Significant differences in immunology between risk groups A. Comparison of score of MHC, EC, SC, CP, and immune cells between high and lwo-risk groups (ns: not significant; *p < 0.05; **p < 0.01; ***p < 0.001); B. Differences of immune score, ESTIMATE score, stromal score, TIDE prediction, Dysfunction prediction, and CD274 prediction between high and low risk groups (ns: not significant; *p < 0.05; **p < 0.01; ***p < 0.001; ****p < 0.0001); C. Several immune checkpoint molecules were significantly different between high risk group and its counterparts.

In order to find out the relationship between immune cells in tumor microenvironment (TME)and progression of OC, we performed the Pearson correlation analysis between immune cells from risk groups based on results which we obtained previously. Our study revealed that CD8 T cells, iDC, and macrophages were significantly positively correlated with risk score (**Supplementary fig. 2A**). We acquired the indicators of immunophenoscore (IPS) on the basis of the expression level of samples from risk groups through the TCLA database. The indicators include MHC (major histocompatibility complex, MHC)molecules, Effector cells (EC), Suppressor cells (SC), and Immunomodulators (CP), and we found that the EC and SC of high-risk group are significantly different from that of low-risk group. We found that there were 17 immune cells with significant difference, such as B cells, CD8 T cells, iDC, Macrophages, and Neutrophils (Fig. 7**A**). Next, we used ESTIMATE algorithm and found that the Immune score, ESTIMATE score and Stromal score of high-risk group are significantly greater than that of low-risk group. TIDE prediction and Dysfunction prediction in high-risk group are significantly greater than that in the low-risk group (Fig. 7**B**). However, CD274 is not significantly different between these 2 groups. Moreover, the results demonstrated expression of immune checkpoints molecules including CD200, CD276, CD46, CD86, HAVCR2, LAIR1, NGFR, PDCD1, PDCD1LG2, TMIGD2, TNFRSF4.Among them, TNFSF4 in high-risk group are significantly different from that of low-risk group (Fig. 7**C**). Overall, there are prominent difference between these 2 risk groups, which suggested that immune-related factors in tumor microenvironment may play an important role in OC progression.

### 2.8 Four expression levels of HRRGs significantly change in the high-risk group

**Fig. 8:**
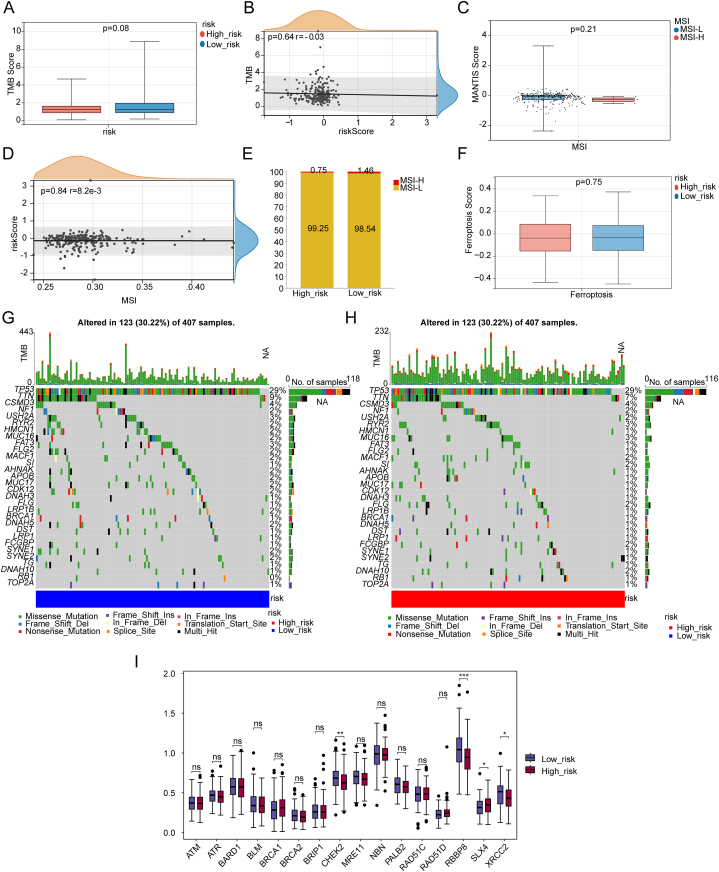
Four HRRG are significantly evolving in the high-risk group A–F: There is no significant difference between 2 risk groups regarding TMB, MSI, ferroptosis. G-H Mutation landscape are almost identical in both risk groups. I: Four HRRG are significantly different expressed between these 2 groups.

In order to explore the effect of potential biomarkers which includes tumor mutation burden (TMB),microsatellite instability (MSI), ferroptosis, and homologous recombination repair genes (HRRGs) in OC, we calculated the TMB score, MSI score, and ferroptosis score of samples from high- and low-risk groups, and found that there is no significant difference between risk groups (Fig. 8A–F). Next, we analyzed the mutation landscape of samples from 2 risk groups and we found that the mutation landscape of high-risk group is almost identical with low-risk group (Fig. 8G and H). Finally, we found that 3 HRRGs which include CHEK2, XRCC2, and RBBP8 are significantly up-regulated and SLX4 is significantly down-regulated in the low-risk group (Fig. 8I).

### 2.9 Drugs effective against OC

**Fig. 9:**
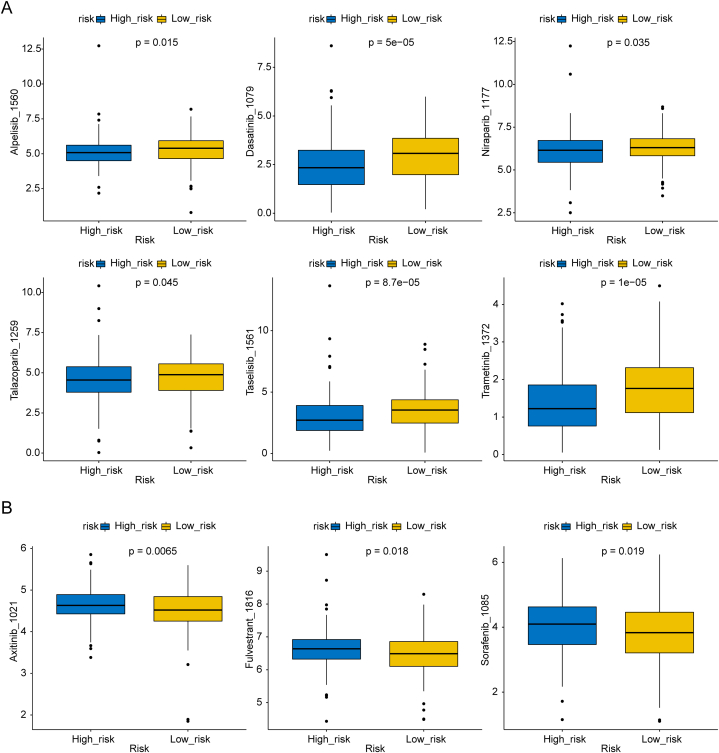
Drugs effective against different groups of OC A.Possibly effective agent against high-risk group OC patients; B. Possibly effective agent against low-risk group OC patients.

We detected the chemotherapy sensitivity of samples by using the GDSC database, and we found that 54 drugs in the high-risk group and 17 drugs in the low-risk group may exert therapeutic effect on OC **(Supplementary table 1)**. In this analysis, high-risk group patients tend to benefit from PI3K inhibitor Alpelisib and Taselisib, classical PARPi Niraparib and Talazoparib; Dasatinib and Trametinib respectively. While, low-risk group patients tend to benefit from VEGFR inhibitor Axitinib, estrogen receptor inhibitor Fulvestrant and RAF inhibitor Sorafenib (Fig. 9A, B).

## 3 Discussion

Malignant metabolism has been a characteristic for cancer, which has attracted attention for decades. In OC, special metabolic characteristics may contribute to chemotherapy resistance and poor survival [12,13].Accumulating evidences have validated that active glutamine metabolism is associated with poor survival in OC and suppressive immunity may be rising in this condition, which will explain poor survival of ovarian cancer from the aspect of TME [14]. In previous study, glutamine metabolism may promote ovarian cancer cell proliferation [15].Glutamine consumption inhibitor may lead to increased overall survival of OC patients [16].Chemotherapy resistance of ovarian cancer may be associated with glutamine metabolism [17], which indicates glutamine metabolism associated target therapy could be quite promising in the treatment of ovarian cancer. However, comprehensive understanding about glutamine metabolism about ovarian cancer is absent in available research nowadays. Here, we try to classify OC patients based on glutamine metabolism via bioinformatic strategies and associated immune state has been discussed in this study.

In our research, we confirmed close relationship between glutamine metabolism and malignant behavior of OC. Some classic signaling pathway were involved in this complicated progress. GMRGs significantly differentially expressed in OC samples when compared with its counterpart**.** Hippo signaling has been in spot light in recent years and this signaling pathway has been uncovered in our study as well. Hippo pathway is an evolutionarily conserved signaling pathway, which is quite important in tissue regeneration, organ development, wound healing epithelial homeostasis and immune modulation [18]. Hippo played vital role in the onset of triple negative breast cancer [19]. In cervical cancer, Hippo-YAP1 signaling could be a major driver in special designed mouse model which develops cervical cancer in situ immediately and this complicated progress may interfere with PI3K pathway activation [20]. Accumulating evidence supported Hippo in malignant transition. In our study, GMRGs-related DEGs between OC and its normal counterpart has been significantly associated with Hippo signaling, which was also compatible with malignant transition of precancerous condition serous tubal intraepithelial carcinoma (STIC) to high grade serous OC. However, the underlying mechanism deserves deep exploration.

Abnormal metabolism played important part in malignancies. Metabolism could be very complicated process. Therefore, it was reasonable that different types of metabolism could interfere with each other, which contributed to metastasis, invasion and TME rebuilding [21]. In our research, Wnt signaling has attracted our attention as well. Wnt has been confirmed associated with development, cell proliferation and metabolism of nutrients such as glucose and amino acid [22,23]. It could crosstalk with other signal pathway [24]. In study of non-malignant condition, YAP/TAZ of Hippo pathway might interfered with Wnt signaling because the former molecules are components of β-catenin destruction complex. The crosstalk between Hippo and Wnt contribute to focused disease, which reminded us to study these 2 pathways as entirety [25]. In our research, GMRGs-related DEGs between OC and its counterparts were closely associated with Hippo and Wnt. This reminds us firstly that these 2 signals might work together in regulation of ovarian cancer glutamine metabolism and secondly crosstalk may frequently happen between these 2 signals in ovarian cancer, which lead to active malignant glutamine metabolism in ovarian cancer.

Homologous recombination repair (HRR)is critical in cancer. Platinum-based chemotherapy is first-line treatment of OC. Hereditary breast and ovarian cancer syndrome (HBOC) is significantly associated with homologous recombination deficiency (HRD) [1]. HRD showed valid relationship with better prognosis of OC. Patients with this molecular character will benefit from platinum containing chemotherapy and PARP inhibitor maintenance therapy [4,26,27]. In our study, we also pay attention to HRD in different types of OC characterized glutamine metabolism. DNA repair and UV response are involved in risk model, which support that glutamine metabolism might be associated with 10.13039/100000171HRD state of 10.13039/100003224OC. In following study, we found that 4 HRR-associated genes significantly differentially expressed between 2 risk groups, which support former results. In canonical study, the mutation associated with loss of function of 4 genes mentioned above will affect homologous repair state. However, the differential expressing of these genes, which could result from meaningful mutation or protein modification in special circumstances such as glutamine metabolism, still needs further study. In pharmacy sensitivity prediction, PARP inhibitor might benefit low risk group patient more, which is compatible with HRD evaluation between these 2 groups. By the way, some promising active agents, which have been widely accepted in other types of malignancies have been confirmed [[28], [29], [30]]. This remind us combinational therapy could be a good choice for treatment and precise decision making can be based on glutamine metabolism.

Immune rebuilding in tumor microenvironment was an important character in malignancies. Cytokines, receptors-ligands and some regulating axis contribute to this process. Recent years, immune checkpoint inhibitors (ICI) become promising in cancer treatment [[31], [32], [33]]. In our findings, 12 kinds of immune checkpoints molecules significantly expressed between high and low risk groups, implying that these genes may regulate immune responses in OC. More and more researches confirmed that TMB, MSI and immune check-point associated molecules expression can predict ICI response in cancer treatment. Poor mismatch match repair may lead to high microsatellite instability (MSI-H), which will lead to better response of ICI therapy possiblely because more tumor antigen present [34].Here, we discussed immune microenvironment of OC in both high and low risk group as well. Immune microenvironment was suppressing one, which will facilitate metastasis and invasion of ovarian cancer. Molecules belonging to PD-1 axis such as CD276, PDCD1, PDCD1LG2 were up-regulated in high risk group, which supports that more obvious suppressing immune microenvironment present in this group. In former part, we discussed HRD of high and low risk groups. HRD may lead to better response of ICI in cancer [35]. However, in comprehensive analysis of our study, MSI and TMB are not significantly different between these 2 groups. This was compatible with the fact that ICI shows modest function in ovarian cancer [[36], [37], [38]]. This result may also imply that the MSI and TMB in this study may not be the only critical factors for risk group categorization or predicting ovarian cancer patients’ outcome. There are many other factors that contribute to risk-rating of patients and treatment response in complex mechanisms associated with tumor development and treatment. Here, we guess ovarian cancer enjoy special immune condition, which has been supported by multi-omic analysis [39]. More treatment strategies were urgently needed. A promising result was obtained in clinical trial DUO-D (combination of classic chemotherapy and PARPi and Avastin), which indicated combinational therapy might break this “cold” immune microenvironment and benefit OC patients.

This study has some limitations. Firstly, experimental validation is absent in this study. Second, we paid more attention on glutamine metabolism. However, metabolism reprogramming is complex progress. Maybe in the future, meta-analysis by informatic methods focusing on crosstalk between glutamine, glucose, even fatty acid will contribute more in ovarian cancer. Despite these limitations, our study firstly constructed risk model involved in ovarian metabolism.

In summary, our research revealed close relationship between glutamine-metabolism and OC firstly.We provided a prognostic prediction model based on these 3 GMRGs. High risk group and low risk group own different biological characters. Besides, we explored the potential tumor immune microenvironment in OC. Inconvenient HRR genes and promising active agent have been discussed as well. Our study should be interpreted carefully due to only a few cases in clinical validation and limited populations of OC patients in open database. However, our findings enrich our understanding of ovarian cancer etiology and TME, which will contribute to individualized therapy during clinical practice and following novel marker research.

## 4 Availability of data and materials

This study obtained open data from the TCGA database (https://cancergenome.nih.gov/), Genotype-Tissue Expression (GTEx) database (https://www.gtexportal.org/), The Cancer Immunome Atlas (TCIA) database (https://tcia/), Genomics of Drug Sensitivity in Cancer (GDSC) database (https://www.cancerrxgene.org/).

## Funding

This study was supported by 10.13039/501100001809National Natural Science Foundation of China (82260,577, 81902652), Yunnan Provincial Medical Reserve Talents Project (H-2019007); Young and Middle-aged Reserve Academic and Technical Leaders in Yunnan Province (CZ0165), Innovative Research Team of Yunnan Province (202305AS350020); Yunnan Province's "Xingdian Talent Support Program" Famous Medical Project (XDYC-MY-2022-0058).

## 5 Methods

### 5.1 Data collection

Data of RNA sequence with survival information of 379 OC patients were downloaded from The Cancer Genome Atlas (TCGA) database (https://portal.gdc.cancer.gov/).Eighty-eight normal patients data were obtained from the Genotype-Tissue Expression (GTEx) database (https://www.gtexportal.org/). GSE119056 microarray including 3 normal and 6 OC samples and GSE140082 microarray including 178 OC samples with total survival information were downloaded from Gene Expression Omnibus (GEO) database (http://www.ncbi.nlm.nih.gov/geo/). Information on Glutamine metabolism-related genes (GMRGs) was gathered from relevant references [40].

### 5.2 Expression pattern and genetic landscape analysis of GMRGs in OC

In order to remove batch effects of RNA expression data obtaining from TCGA and GTEx databases, R package “bladderbatch” (version 1.34.0) was performed. These data were used to detect the different expression of GMRGs between normal and OC samples. Next, protein-protein interaction (PPI) network of GMRGs was created for the detection of reciprocity among GMRGs by using the STRING database. Besides, R package “maftools” (version 2.12.0) was applied to calculate mutations of GMRGs based on somatic mutation data [41]. Moreover, the copy number variation (CNV) and the position of GMRGs in chromosome were identified.

### 5.3 OC samples were divided into different clusters based on GMRGs

Univariate Cox analysis was used to assess whether GMRGs are associated with survival by R package “survival” (version 3.2–13) [42]. In this process, gene expression was compared with survival time as a continuous variable in order to filter GMRGs according to *p* < 0.05. The genes remained would work in forest plot with the help of R package “forestplot” (version 2.0.1). After, ssGSEA in R package “GSVA” (version 1.40.1) was performed to obtain scores of OC patients based on the expression level of genes screened out in previous Univariate Cox analysis [43]. Thereafter, OC patients were divided into different clusters based on the median of the score.

To clarify the survival differences between different clusters, survival analysis was performed by R package “survminer” (version 0.4.9). After, R package “GSVA” was utilized to perform ssGSEA based on 50 hallmark pathways. Heatmap was created by R package “pheatmap” (version 1.0.12) [43] at the same time. Meanwhile, the ssGSEA was used to deduce the percentage of infiltrating immune cells in samples from different clusters on the basis of a gene set that includes 24 immune cell markers, and the rank sum test was performed during significance analysis (*p* < 0.05). R package “ggplot2” (version 3.3.5) was adopted to produce a violin plot [44]. Finally, we got TIDE prediction, CD274 prediction, T cell dysfunction prediction, and T cell exclusion prediction of samples by the Tumor Immune Dysfunction and Exclusion (TIDE) database (http://tide.dfci.harvard.edu/) [44].

### 5.4 Differentially expressed gene analyses between normal and OC samples

In order to obtain DEGs between 88 samples of normal patients from the GTEx database and 379 samples from TCGA, R package “limma” was performed and the screening parameters were *adj.p* < 0.05 and |log2FC| > 0.5 [45]. We did the same in other 2 pairs (the first pair: 3 normal and 6 OC patients samples from GSE 119056 microarray; the second pair: clusters of OC).R package “ggplot2” and “pheatmap” were utilized to create volcano plot and heatmap plot, respectively. After, these 3 kinds of DEGs were intersected to obtain a GMRGs-related DEGs set. Moreover, we distributed the OC samples again with the same methods which includes Univariate Cox analysis and survival analysis [45].

### 5.5 Enrichment analysis of GMRGs-related DEGs

We performed enrichment analysis of GMRGs-related DEGs to find enriched functions and pathways by using R package “clusterProfiler” (version 4.0.2) based on Kyoto Encyclopedia of Genes and Genomes (KEGG) and Gene ontology (GO) [46]. The significant thresholds are *adj.p* < 0.05 and count ≧ 1. GO includes biological process (BP), molecular functions (MF), and cellular components (CC).

### 5.6 Risk model construction and validation

Data from TCGA and GTEx were used as training sets. Data from GSE140082 were adopted as validation set. The Least absolute shrinkage and selection operator (LASSO) regression is a regularization technique used for prediction more accurately than regression methods, and it is widely used in fields with large datasets such as genomics [47]. Here, we have applied R package “glmnet” (version 4.1-3) during LASSO in order to further filtering genes sifted out by Univariate Cox analysis previously [48]. Next, predict.coxph function in R package “survival” was adopted to calculate risk score according to formula: *risk score = β1×X1+β2×X2+ … +βn×Xn*, in which β represents the regression coefficient, and the Hazard Rati value (HR) can be obtained after taking anti-log for it [42]. Finally, we performed survival analysis. Risk curves and ROC curves based on both training set and validation set were generated to estimate and verify the performance of the risk model.

### 5.7 Enrichment analysis of risk model

In order to explore which pathways are activated in the high- and low-risk group, we download the hallmark gene set via R package “msigbr” (version 7.4.1), and then performed the GSVA analysis and variance analysis. The filtering parameters were *p* < 0.05 and |t | > 2. Next, we enriched pathways in risk groups by R package “GSVA”. Finally, we analyzed correlation between risk score and enrichment of pathways via R package “corrplot” (version 0.91).

### 5.8 Tumor microenvironment in risk model

Tumor microenvironment (TME) significantly affects diagnosis, survival outcomes, and clinical outcomes of OC. Infiltrating immune cells are important components of TME. Therefore, we performed Pearson correlation analysis based on previous results in order to confirm relationship between risk score and percentage of immune cells in samples. Following, we adopted the rank sum test to analyze the significant difference among immune cells in samples from 2 risk groups (*p* < 0.05), and visualized the results were obtained via R package “ggplot2” [44]. Then, we used ESTIMATE algorithm to deduce the proportion of stromal cells and immune cells in samples from risk groups. In this part, we paid more attention to three scores: Immune score, ESTIMATE score, and Stromal score.

Tumor mutational burden (TMB), Microsatellite Instability (MSI), and Homologous recombinant repair genes in risk model.

TMB is an OC biomarker and MSI is a phenomenon of the length change of MS sequence because of insertion or mutation occurs when DNA replication and MSI is frequently caused by a defect in the mismatch repair (MMR) function. TMB score, MSI score, and mutation landscape of each sample from risk groups were calculated by R package “maftools”.Significance analysis was performed via rank sum test (*p* < 0.05) [41]. Moreover, we obtained 483 regulator factors or maker genes of ferroptosis from the Ferroptosis database (FerrDB) to calculate the ferroptosis score of samples from risk groups via ssGSEA in R package “GSVA” [43]. Significance analysis was performed by the rank sum test (*p* < 0.05). Finally, we checked expression level of homologous recombinant repair genes (HRRGs) in samples.

### 5.9 Chemotherapy sensitivity in the risk model

Moreover, immunophenscore (IPS) was obtained from The Cancer Immunome Atlas (TCIA) database (https://tcia/) on the basis of the expression level of samples. Next, TIDE prediction, CD274 prediction, T cell dysfunction prediction, and T cell exclusion prediction of samples were obtained from the TIDE database. Besides, we download the drug list from Genomics of Drug Sensitivity in Cancer (GDSC) database (https://www.cancerrxgene.org/) to evaluate the drug sensitivities of samples from risk models. We utilized R package “oncoPredict” (version 0.2) to calculate IC50 in different groups, which illustrates the curative effect of drugs [49].

## CRediT authorship contribution statement

**Xie Yaqing:** Writing – original draft, Resources, Data curation. **Gao Yang:** Methodology, Formal analysis. **Yang Linlin:** Validation, Methodology. **Ruan Youqing:** Visualization, Investigation, Formal analysis. **Yang Henghui:** Software, Methodology. **Yang Ping:** Validation, Methodology. **Yang Hongying:** Writing – review & editing, Validation. **Shaojia Wang:** Writing – review & editing, Supervision, Funding acquisition, Conceptualization.

## Declaration of competing interest

The authors declare that they have no known competing financial interests or personal relationships that could have appeared to influence the work reported in this paper.

## Figures and Tables

**figs1:**
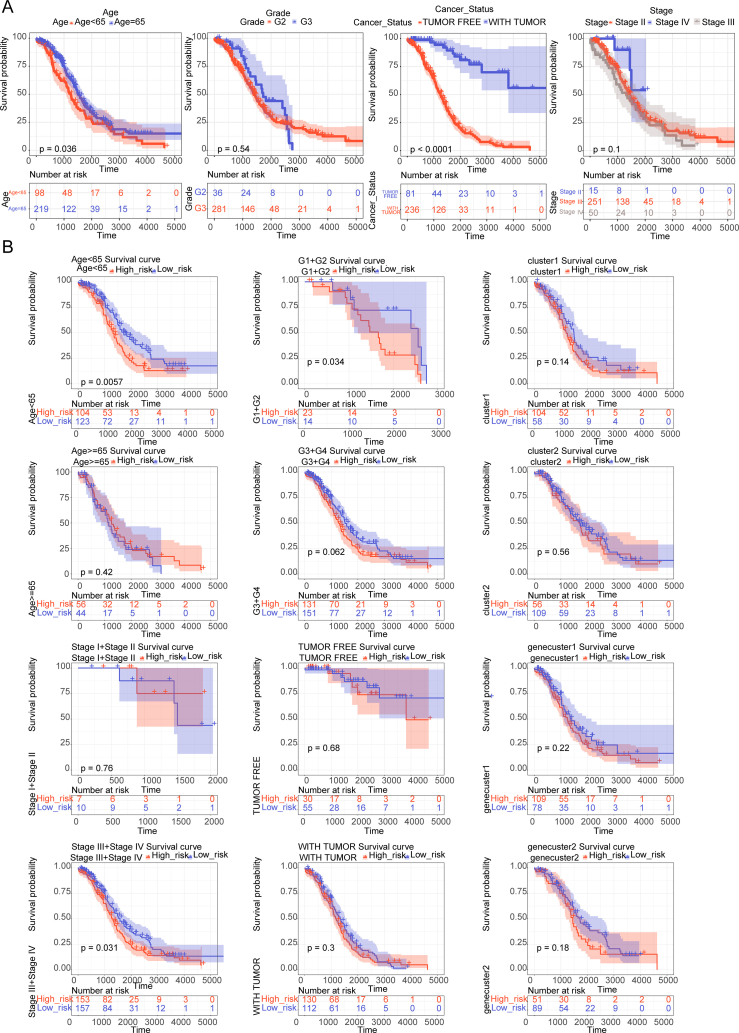


**Figure:**
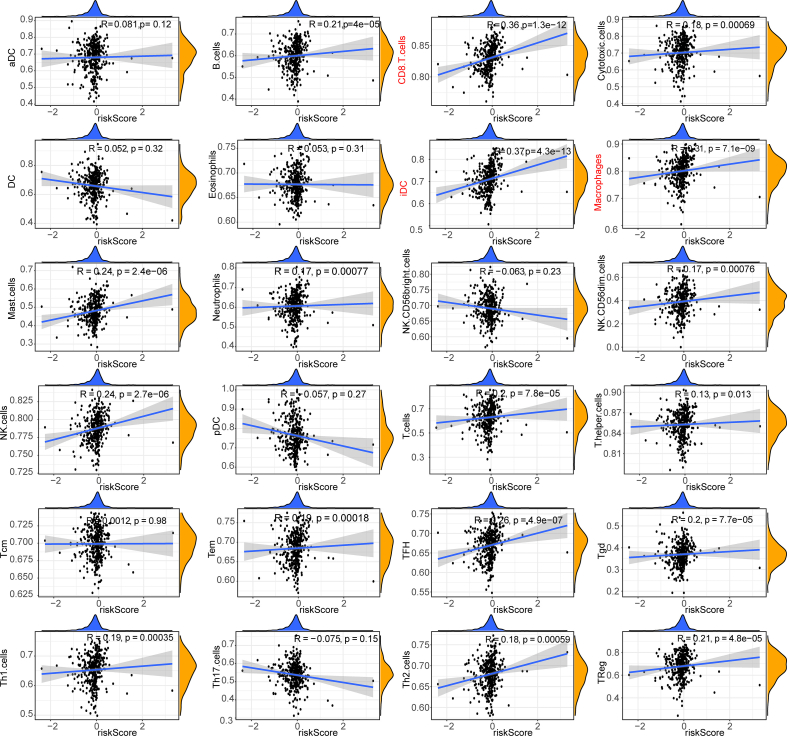
figs2
